# Endometriosis presenting as an acute groin swelling: a case report

**DOI:** 10.4076/1757-1626-2-6438

**Published:** 2009-08-12

**Authors:** Catherine L Boereboom, Nicholas FS Watson, Rangsamy Sivakumar, Gurprit Atwal, Gillian M Tierney

**Affiliations:** 1 Department of Colorectal Surgery, Derby City General HospitalUttoxeter Road, Derby, Derbyshire, DE22 3NEUK; 2 Department of Pathology, Derby City General HospitalUttoxeter Road, Derby, Derbyshire, DE22 3NEUK

## Abstract

**Introduction:**

Many conditions present as groin swellings, in both the elective and emergency setting. The management of these conditions varies widely, thus a prompt and accurate diagnosis is important.

**Case presentation:**

A 27 year old female presented with an acute painful swelling in her right groin. A preliminary diagnosis of an incarcerated femoral hernia led to urgent surgical exploration. Histology of the excised tissue showed appearances consistent with endometriosis.

**Conclusion:**

Endometriosis is an unusual cause of an acute groin mass, which should be considered as a differential diagnosis in women of childbearing age.

## Introduction

Endometriosis is a condition in which foci of endometrial glandular tissues are found beyond the uterine cavity. It affects approximately 12% of women and commonly involves the ovaries, rectovaginal pouch and the pelvic peritoneum. Rarely, foci of endometriosis have been found in bowel, the umbilicus, abdominal surgical scars and in the lungs.

In many cases endometriosis is asymptomatic. However, the commonest presenting symptom is of cyclical pelvic pain. At affected sites outside the pelvis cyclical bleeding may also be noticed. Laparoscopic evaluation of the abdominal cavity is often used to distinguish endometriosis from other conditions causing chronic pelvic pain such as chronic pelvic inflammatory disease, pelvic congestion syndrome and interstitial cystitis.

Suppression of ovarian function for six months has been shown to reduce endometriosis-associated pain. Combined oral contraceptives, danazol, gestrinone, medroxyprogesterone acetate and GnRH agonists are equally effective but have differing side effect profiles and costs [1]. Surgical treatment options include excision or diathermy ablation of the ectopic tissue, with total hysterectomy and bilateral salpingo-oophorectomy being the most radical treatment.

Although first reported in the British Medical Journal in 1949 [2], endometriosis occurring in the inguinal canal or attached to the round ligament is very uncommon. It classically presents as a groin mass and can coincide with other pathologies such as inguinal hernias, raising diagnostic difficulties [3]. Patients may complain of a mass occurring in a cyclical manner, and serial MRI scanning has been used to demonstrate that the mass may vary in size with the patients’ menstrual cycle [4]. An ultrasound scan may support the diagnosis but appearances can be similar to an inguinal hernia [5]. In cases where imaging has been non-conclusive fine needle aspiration cytology has been used to establish a firm diagnosis prior to surgery [6]. Surgical excision is indicated to both confirm the diagnosis and prevent further episodes of pain. Intra-abdominal disease should be assessed in symptomatic patients. Although it is common for patients with endometrial deposits on the round ligament to also have intraperitoneal disease this is not always the case. The spectrum ranges from those with round ligament disease alone to those with rudimentary uterine tissue contained within the groin mass [7]. Laparoscopy is the gold standard diagnostic test but carries its own risks [1].

## Case presentation

A 27 year old nulliparous white Caucasian woman presented to the surgical admissions unit with a two day history of an acutely painful swelling in her right groin. The swelling was tender to touch, and her pain was made worse by lying down.

The patient was previously fit and well, a non-smoker, and her past medical history included irritable bowel syndrome and lactose intolerance. Her current medication was a combined oral contraceptive pill.

On examination a tender, 5 × 2 cm, non-compressible, non-reducible ovoid swelling in the right groin was noted and a diagnosis of either an incarcerated femoral hernia or an enlarged lymph node was made.

**Figure 1.**
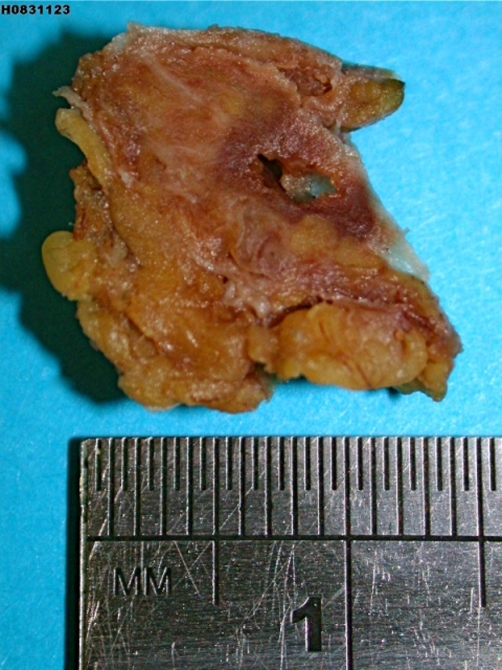
Cross section through of fibro-fatty tissue showing a cystic space with surrounding haemorrhage.

Surgical exploration of the groin was carried out the same day under general anaesthetic. On incising the skin and subcutaneous fat, the operating surgeon was able to palpate the mass deep to the superficial inguinal ring. On opening external oblique to access the inguinal canal, a hard inflammatory mass was found at the insertion of the round ligament into the pubic tubercle (Figure 1). There was no evidence of a coexisting inguinal or femoral hernia, nor any loco-regional lymphadenopathy. The mass was excised and sent for histological examination.

The patient made an uncomplicated recovery from her operation and was discharged home the following day.

**Figure 2.**
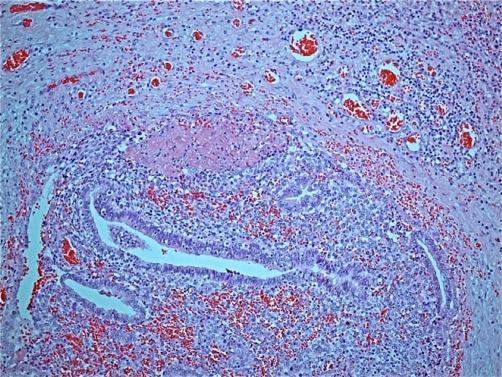
Haematoxylin and eosin stained section showing a focus of endometriosis with associated haemorrhage and surrounding inflamed fibrous tissue, x100 original magnification.

**Figure 3.**
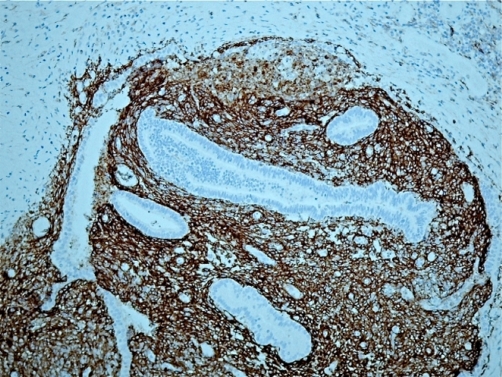
Immunohistochemical staining with anti-CD10 antibody demonstrating positive reaction in areas of endometrial stroma, x100 original magnification.

Histology of the excised tissue reported fibroadipose tissue showing foci of endometrial type glandular epithelium and stroma which stained positive with anti-CD 10 antibody (Figures 2 and 3). This was associated with haemorrhage, fat necrosis, focal fibrin deposition and haemosiderin-like pigment. The appearances were consistent with endometriosis.

On subsequent outpatient review the surgical wound had healed without complications and the patient had no further symptoms.

## Conclusions

The differential diagnosis of a swelling in the groin is broad. Common causes are inguinal or femoral herniae, enlarged lymph nodes, swellings arising from structures within the skin such as lipomata and sebaceous cysts, and vascular pathology such as saphena varix and femoral artery aneurysms. Ectopic and incompletely descended testes may also be located in the groin.

This case illustrates an unusual cause of an acute painful groin swelling in a young woman - endometriosis. The timely diagnosis of this condition allowed optimal patient management. It acts as a reminder that although common conditions will form the mainstay of our surgical practice we must be also be alert to the more extra-ordinary presentations.
